# Monostotic Fibrous Dysplasia of the Mandible in a 9-Year-Old Male Patient Treated with a Conservative Surgical Treatment: A Case Report and 15-Year Follow-Up

**DOI:** 10.1155/2021/9963478

**Published:** 2021-05-03

**Authors:** Antoine Berberi, Georges Aoun, Emile Khalaf, Georges Aad

**Affiliations:** ^1^Department of Oral and Maxillofacial Surgery, Faculty of Dental Medicine, Lebanese University, Lebanon; ^2^Department of Oral Medicine and Maxillofacial Radiology, Faculty of Dental Medicine, Lebanese University, Lebanon; ^3^Beirut Arab University, Lebanon

## Abstract

Fibrous dysplasia is a developmental disorder of the bone that originates from a genetic defect disturbing the osteogenesis leading to the replacement of normal bone with the excess proliferation of fibrous tissue. It can be associated with hyperpigmentation of the skin and endocrine disorders. Fibrous dysplasia can manifest in a monostotic form affecting one bone or in a polyostotic form involving several bones. Approximately 30% of monostotic forms are observed in the maxilla and the mandible. It frequently appears in the posterior region and is usually unilateral. It is found in teenagers and could become static after adulthood. Patients can present with swelling, facial asymmetry, pain, or numbness on the affected side. Treatment modalities vary between conservative surgical treatment, radical surgical approach, and medical treatment based on bisphosphonates. Here, we present a case of a monostotic form of fibrous dysplasia affecting the posterior left region of the mandible in a 9-year-old male complaining of gradually increased swelling on the left mandibular side of one-year duration. The diagnosis of fibrous dysplasia is established based on clinical, radiographical, and histopathological features. Conservative surgery is implemented with surgical shaving and reencountering of the bone excess to reduce the facial asymmetry. Recurrence is reported 10 years later and is also treated with a localized osteoplasty and remodeling of the bone contours. Five years later, the lesion remains stable. In conclusion, a conservative approach should be adopted as the first line of treatment for young patients suffering from monostotic fibrous dysplasia.

## 1. Introduction

Fibrous dysplasia (FD) is a benign intraosseous tumor-like process, where the medullary bone is substituted by fibrous connective tissue leading to undeveloped and inadequately calcified bone [1–4]. FD involves an inequity among osteoblastic and osteoclastic activities triggered by a genetic mutation in the stimulatory G protein (GS*α*) [5].

The increase in the activity of GS*α* in osteoblast progenitor cells and osteoclasts is stimulated by the amplified production of interleukin-6 stromal cells [5–7].

According to the current World Health Organization classification of bone tumors, FD belongs to tumors with an undefined neoplastic nature [4]. FD can be associated with hormonal dysregulations, most frequently precocious puberty with cutaneous manifestations seen in the McCune-Albright syndrome, MAS, or intramuscular myxomas in Mazabraud's syndrome, MS [1, 2]. FD commonly develops in the first or second decades of life, affects women more than men with a ratio of 2 : 1, and is usually asymptomatic with very low evolution [1, 2]. FD occurs either as a monostotic form affecting a single bone or as a polyostotic form spreading into several bones [1–4]. Monostotic fibrous dysplasia (MFD) is around 10 times more common than polyostotic fibrous dysplasia (PFD) and is frequently unilateral [3, 4]. The lack of stable bone matrix formation can manifest in a multitude of ways, involving facial distortion and asymmetry [5–7]. In the jaws, tooth movement, occlusion problems, pain, paresthesia, and interference with dental eruption can be observed [3]. Rapid growth of the bone lesion as well as an increase in the level of alkaline phosphatase could be indicators to possible malignant transformation [2].

In this manuscript, we report a case of MFD in the mandible of a 9-year-old male patient. The diagnosis is established based on different imaging techniques and confirmed with biopsy. Surgery for bone remodeling to correct the patient's facial contour is the treatment adopted.

## 2. Case Report

A 9-year-old male presented to our clinics with his parents complaining of swelling and asymmetry in the left side of his mandible.

The questionnaires revealed that the expansion of the swelling was slow, gradually increasing during the past year with no pain reported.

**Figure 1:**
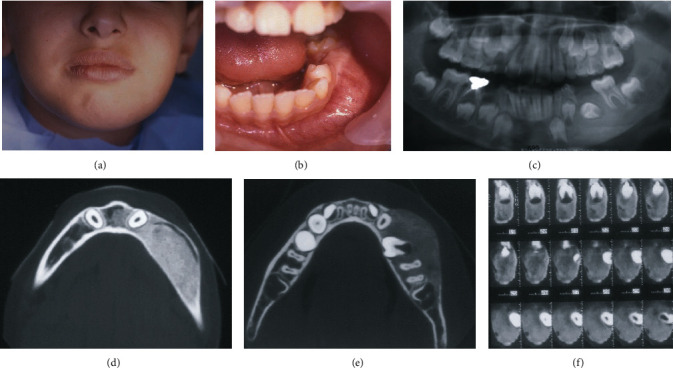
(a) Facial asymmetry on the left side. (b) Swelling in the buccal side of the mandible. (c) Panoramic X-ray showing the radiopaque image englobing the dental germ of the permanent second premolar. (d) The course of the mandibular canal displaced buccally is shown on the axial image of the CBCT. (e) Axial image of the CBCT showing the expansion of the body of the mandible. (f) Para-axial images of the CBCT revealed the ossification of the lesion and the positions of the retained dental germ and the mandibular canal.

Extraoral examination showed a mild facial asymmetry associated with a well-defined swelling on the left side of the mandible measuring 3 to 4 cm (Figure 1(a)). No cutaneous pigmentations on the face, no limitations of mouth opening, and no enlarged submandibular lymph nodes were noticed.

Intraoral examination displayed expansion of the buccal cortical plate, extending from the mandibular left incisal to the first molar, and the first deciduous molar was absent (Figure 1(b)). On palpation, it was hard in constancy, fixed, with no signs of mucosal inflammation observed and no signs of paresthesia.

A panoramic radiograph exposed the loss of trabecular structure and a radiopaque image showing a “ground-glass” feature was observed on the left side of the mandible englobing the dental germ of the permanent second premolar (Figure 1(c)).

The cone-beam computed tomography (CBCT) axial images revealed a radiopacity lesion with an expansion and thinning of the buccal bone cortical with well-defined borders (Figure 1(d)). The dental germ was in the middle of the lesion (Figure 1(e)), and the para-axial images showed the mandibular canal displaced buccally and toward the lower border of the mandible (Figure 1(f)).

Based on the evaluation of the clinical and radiological data, a provisional diagnosis of FD was made, and differential diagnoses of MAS, MS, and hyperparathyroidism were considered.

As the germ needed to be removed, we discussed with the parents the surgical procedure required to extract the germ along with the need for an osteoplasty to remove the excess bone and a biopsy to be taken during the procedure.

**Figure 2:**
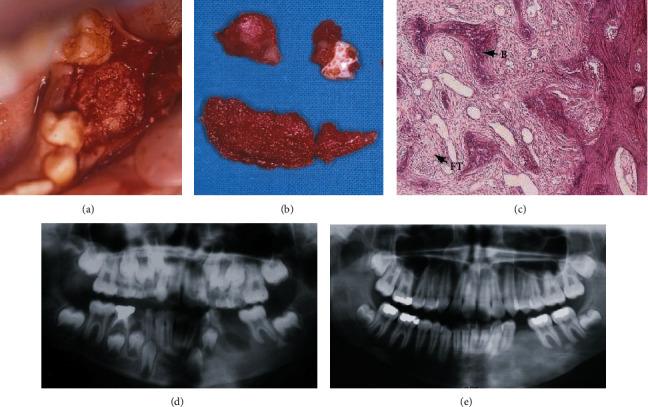
(a) Clinical view of the excess bone. (b) The excised bone with the dental germ. (c) HE × 10 showing irregularly shaped trabeculae of immature bone within proliferating fibroblastic tissue (B: bone; FT: fibrous tissue). (d) One-week panoramic radiograph. (e) Five-year panoramic radiograph.

Under locoregional analgesia, a conservative surgical excision of the buccal bone was proceeded, with extraction of the dental germ and remodeling of the region disturbed by the asymmetry (Figure 2(a)). Macroscopically, the excised bone was soft with spongy aspects (Figure 2(b)). The histological results were consistent with FD. The hematoxylin-eosin staining revealed irregularly shaped trabeculae of immature bone within proliferating fibroblastic and vascularized stroma (Figure 2(c)).

The patient was advised to consult his pediatrician for further clinical and radiological investigations. All the laboratory tests and radiological images were in favor of localized FD mandibular lesions.

The patient was followed up yearly, both clinically and radiologically with a panoramic radiograph (Figures 2(d) and 2(e)).

**Figure 3:**
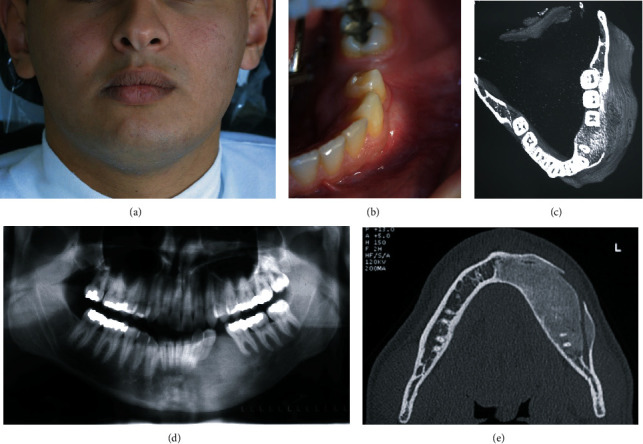
(a) Facial asymmetry at 10 years. (b) Clinical view of the intraoral swelling. (c) Panoramic X-ray at 10 years. (d) Axial image of a CBCT showing the expansion of the lesion to the midline and discontinuity of the buccal cortex. (e) 3D reconstruction showed an increase in the volume of the left hemimandible.

Ten years later, the patient complained of left mandibular asymmetry (Figure 3(a)).

Clinical examination revealed a lingual cortical bone expansion beside the buccal one (Figures 3(b) and 3(c)).

Panoramic X-ray showed the same image of a radiopaque area with the appearance of ground glass aspects in the left hemimandible (Figure 3(d)).

The axial images of the CBCT presented a discontinuity of the buccal cortex, expansion of the lingual one, and expansion of the lesion anteriorly to the middle line of the mandible.

The mandibular canal was still displaced to the lower border of the mandible (Figure 3(e)).

A 3D reconstruction showed an increase in the volume of the left hemimandible, confirming mandibular asymmetry (Figure 3(f)).

**Figure 4:**
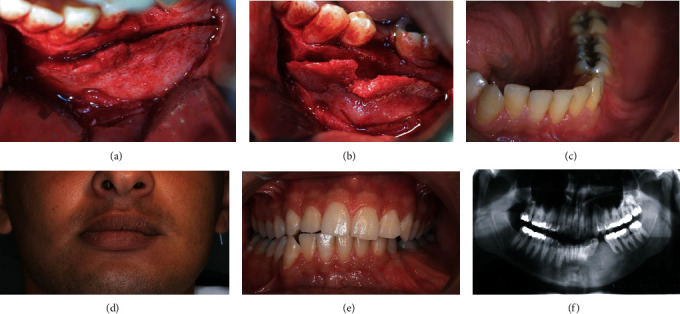
(a) Intraoperative view for the surgical approach. (b) The excised bone with respect to the mental foramen. (c) Healing of the soft tissue one month later. (d) Facial stability after 5 years following the second surgery and 15 years after the first surgery. (e) Clinical appearance of the soft healing after 15 years of the first surgery. (f) Panoramic X-ray after 15 years of the first surgery.

After discussing the findings with the patient, a second surgery was decided. Under locoregional analgesia, a wide osteoplasty was performed, and the buccal bone was reshaped until the limit of the mental nerve anteriorly and the position of the mandibular canal apically (Figures 4(a) and 4(b)). The histology results confirmed the previous one.

No complications were recorded in the follow-up period, and the patient was satisfied with the esthetic results (Figure 4(c)).

Five years later the clinical and radiological exams showed a stabilization of the lesion (Figures 4(d)–4(f)).

## 3. Discussion

FD represents 7% of all benign bone tumors and includes the facial bones in approximately 10%–27% of MFD patients [1–4]. Chen et al. state that MFD affects the maxilla more regularly than the mandible [8]. Kruse et al. reveal that equal distribution between the maxilla and the mandible exists [9]. Lee et al. show that the mandible is touched in 19 cases and the maxilla in 6 [10]. Davidova et al. report that maxilla (23/38) is more involved than the mandible (15/38) [11].

The differential diagnosis of FD comprises simple bone cyst, nonossifying fibroma, osseous-fibrous dysplasia, adamantinoma, low-grade intramedullary osteosarcoma, Paget's disease, MAS, and MS [1, 10, 11].

The CBCT offers complete information about the bone lesions associated with the FD, its border, and the exact relation with the anatomical elements [8].

Treatment modalities of FD such as conservative surgery [9, 10, 12, 13], radical excision [14, 15], and medical treatment with bisphosphonates [16, 17] are suggested. Conservative surgical management implicates surgical excision of the poorly mineralized bone followed by a modeling osteoplasty through an intraoral approach which can potentially help with the histological confirmation of the diagnosis [9, 10, 12, 13]. The surgical treatment should take into consideration the age of the patient [4, 9].

The recurrence of fibrous dysplasia is reported in relation to puberty or skeletal maturation; however, its evolution is constant into adulthood [1, 4, 9].

Kruse et al. describe the treatment of MFD in 8 patients with a modeling osteotomy with good results [9]. Valentini et al. state that, in a retrospective study involving 95 patients with FD situated in the craniomaxillofacial area, the following proportions are seen: 76% MFD, 22% PFD, and 2% McCune-Albright syndrome. Of 95 patients, surgical treatment is done for only 68 patients (radical removal of the affected bone for 62 patients and conservative treatment for 6 patients); the remaining patients refused surgery although the authors suggest that radical surgery avoids recurrence [15].

The use of bisphosphonates to decrease osteoclastic activities is considered as a treatment option apart from surgery [16, 17]. Intravenous, not oral, bisphosphonates can be considered in the treatment of persistent, moderate to severe bone pain [18]. Lala et al. state that the use of bisphosphonates improves the clinical situation in children and adults [17]. To note, osteonecrosis of the jaws is a major complication of bisphosphonate treatment [18, 19]. Thus, caution should be considered when prescribing bisphosphonates for a long period in patients suffering from FD [19].

In our case, the patient is young, and the diagnosis is based on clinical, radiological, and histological data. Since the asymmetry is disturbing to the patient, conservative surgical approach is adopted keeping in mind that he is 9 years old, around puberty with a skeletal system that is still in a growth period. At this age, radical excision is very traumatic and can affect the facial growth of the patient. Also, the use of bisphosphonates does not significantly impact the age-dependent decrease in bone turnover, and it does not prevent the progression of FD disease burden in young as stated by Florenzano et al [18]. The CBCT images are very useful to delimitate the lesions, especially when near the mental foramen in the mandibular canal.

Recurrence is observed ten years later, and bone remodeling is adopted for the reasons previously mentioned. Five years later, the clinical situation is stable, and the patient remains satisfied with the esthetic results.

## 4. Conclusion

Conservative treatment for MFD should be considered, particularly if the patient is young. Localized surgical excision of the demineralized bone followed by an osteoplasty and contour remodeling is one of the most conservative approaches for the treatment of asymmetry. Observation and follow-up should be maintained closely.
